# A Rare Case of Metastasis of Renal Clear Cell Carcinoma to the Thyroid Gland, Presenting as a Goiter Nodule, Three Years After Nephrectomy

**DOI:** 10.30699/ijp.2020.117839.2282

**Published:** 2020-07-16

**Authors:** Hedieh Moradi Tabriz, Arezoo Eftekhar Javadi, Atieh Zandnejadi

**Affiliations:** 1 *Department of Anatomical and Surgical Pathology and Laboratory Medicine, Sina Hospital, Tehran University of Medical Sciences, Tehran, Iran*

**Keywords:** Renal Cell Carcinoma, Thyroid Gland, Metastasis, Multinodular Goiter

## Abstract

Thyroid gland metastatic tumors are rare in clinical practice. Clear cell RCC is one of common metastatic tumors to thyroid. We here reported a case of incidentally found clear cell renal carcinoma metastasis to the thyroid gland 3 years after nephrectomy, in the thyroidectomy procedure performed for the patient due to the thyroid enlargement caused by multinodular goiter. A 65-year-old Iranian man with a history of multinodular goiter referred to our surgery clinic for thyroidectomy because of compressive effects on the trachea. Patient had a history of nephrectomy due to clear cell RCC 3 years ago. After thyroidectomy, gross and histological examination of thyroid revealed clear cell renal carcinoma metastasis to the thyroid gland in the setting of a multinodular goiter. The diagnosis was confirmed by immunohistochemistry staining. Patients with multinodular goiter are more prone to present with metastasis to thyroid gland if they have a history of malignancy, especially renal cell carcinoma.

## Introduction

Renal cell carcinoma is the most common type of kidney cancer. RCC has unpredictable and diverse behavior and is called “internist’s tumor” (1). Approximately one-third of patients will have metastatic disease at diagnosis and a quarter of patients will develop metastasis after curative-intent nephrectomy (2).

Bone, lymph nodes, lungs, and brain are expected as metastatic sites, however metastasis may be found at unusual locations such as skin, testis, maxillary antrum, and cervix (3). Metastasis to thyroid gland is relatively rare despite its rich blood supply; but RCC is one of the most common types of neoplasms for metastasis to the thyroid gland. Clear cell variant of RCC has tendency to metastasize widely, even before local symptoms (4). Herein we reported a case of clear cell type RCC metastasis to thyroid gland 3 years after nephrectomy as an incidental finding after thyroidectomy due to a multinodular goiter.

##  Case Report

A 65 year-old male patient was referred to Sina hospital (Tehran, Iran) for hyperthyroidism checkup. He had a history of hyperthyroidism with methimazole treatment. He had also a history of nephrectomy due to renal cell carcinoma clear cell type 3 years prior. (PT3a). The renal tumor had invaded to the sinus adipose tissue and extended to the Gerota’s adipose tissue but surgical margins and renal vein were free of involvement. He had a history of radical orchiectomy two years prior with a diagnosis of chronic granulomatous epididymo-orchitis and possibility of a burnt out tumor due to scar tissue.

The thyroid enlargement from many years ago showed progression and in the last ultrasonography report, both thyroid lobes demonstrated enlargement with severe heterogenic parenchymal echogenicity and also many hypo-echo thyroid nodules with micro-calcification seen in both lobes. The radiologist also noticed two largest nodules; one hypo echo nodule M: 38 * 26 mm and the other was spongy form nodule M: 24* 17 mm in the right thyroid lobe.

CT scan of cervicothoracic region revealed remarkable thyroid enlargement with extension to the thoracic inlet with compressive effects on the adjacent trachea in favor of a multinodular goiter. 

**Fig. 1:**
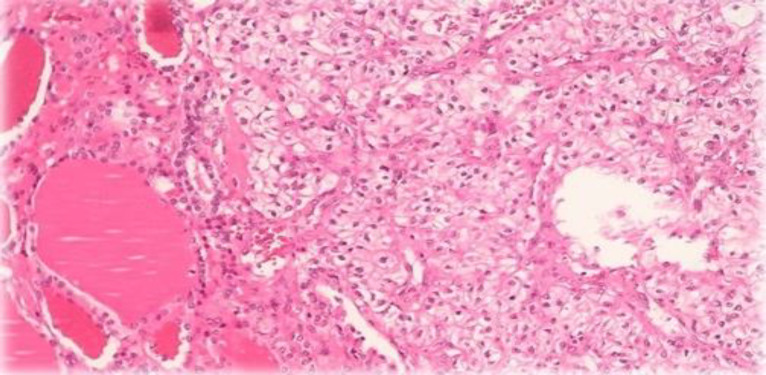
Microscopic view of renal cell carcinoma, Clear cell type metastasis into thyroid gland

**Fig. 2:**
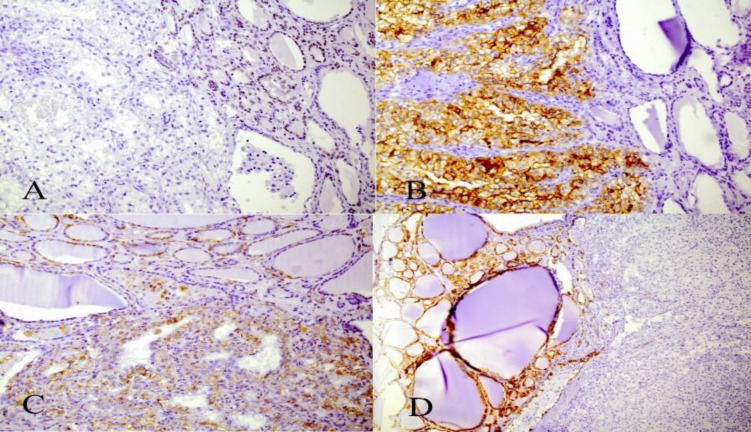
IHC staining of nodule for TTF1 marker shows positivity in thyroid follicular cells, negative in metastatic cells (A); CD10 staining shows positivity of metastatic cells, normal thyroid follicular cells are negative (B); Vimentin staining shows positivity in metastatic cells (C); CK7 staining shows positivity of thyroid follicular cells and negative results in metastatic cells (D).

The patient was referred to a surgeon. After thyroidectomy, the thyroid gland was inspected in our pathology department. We found enlarged multinodular thyroid gland with a soft fragile yellowish nodule M 25 * 20 mm in superior part of the right lobe as an incidental finding. Microscopic evaluations showed metastatic clear cell renal cell carcinoma into the thyroid right lobe. Other portions showed features of a multinodular goiter (Figure 1). We confirmed our diagnosis by IHC which showed positive results for CD10, PAX8 and Vimentin and negative for CK7, CK20 and TTF-1 (Figure 2). The patient was referred to an oncologist for further evaluation and treatment.

## Discussion

Thyroid gland metastasis tumors are very rare in clinic. The most common tumors with metastasis to thyroid glands are of breast, lung and skin and kidney primaries, except for lymphoma, leukemia and malignant melanomas (5,6). Diagnosis of metastatic renal cell carcinoma within thyroid gland is challenging using clinical manifestations and radiologic evaluation dut to the non-specific findings. FNA biopsy and immunohistochemistry staining are more helpful (7).

Some Radiologic features include an oval-shaped hypoechoic solid nodule with well-defined smooth margins and increased vascularity on thyroid ultrasonography. These features with a medical history of RCC should raise clinical suspicion (8).

It seems that FNA biopsy is a good procedure to approach a suspicious nodule in patients with history of renal cell carcinoma because of its low morbidity and expenses. Unfortunately, FNA biopsy has a high rate of false negative results up to 28.7%; so when FNA biopsy taken from a nodule has negative or suspicious results, the possibility of a malignancy still should be taken in consideration. (9).

The thyroid gland has predisposition for metastasis due to the metabolic changes such as decrease in oxygen and iodine contents for example in goiter and thyroiditis (10,11).

In 2019 Khairul-Asri and* et al.* reported two cases of RCC metastasis to the thyroid gland presenting as thyrotoxic goiter and asymptomatic thyroid nodule (12); similar to what was seen in our case. These cases should aware clinicians to consider metastatic RCC as a differential diagnosis in approaching a new thyroid mass or nodule.

Symptoms of metastasis can vary from no manifestation to even dysphagia and hypercalcemia in some cases (3). Autopsy investigations of patients with history of metastatic malignancies have suggested that thyroid metastases are under-diagnosed in up to 20% of patients (13).

As in our case, the patient had no sign or symptom, but a thyroid enlargement in the setting of a multinodular goiter and the metastasis was detected through routine examination and follow-up. . 

Ultrasonographic studies of thyroid gland combined with cytological findings obtained via FNA of of thyroid nodules, and previous medical history of a RCC can provide earlier diagnosis of RCC metastasis before thyroidectomy consideration (14).

Metastasis from Renal cell carcinoma can be found more than 20 years following nephrectomy (15). Continued follow up and thyroid examination in patients with a history of RCC following nephrectomy would be recommended particularly in those patients with some underlying conditions such as multinodular goiter and thyroiditis.

## Conclusion

In patients with a medical history of RCC, especially clear cell variant, a thyroid nodule even in background of benign thyroid diseases such as adenomatous goiter, should prompt work-up for metastasis.
